# Effect of surgeon-related factors on outcome of retinal detachment surgery: analyses of data in Japan-retinal detachment registry

**DOI:** 10.1038/s41598-022-07838-5

**Published:** 2022-03-10

**Authors:** Keita Yamakiri, Taiji Sakamoto, Chihaya Koriyama, Ryo Kawasaki, Takayuki Baba, Koichi Nishitsuka, Takashi Koto, Hiroto Terasaki, Japan Retinal Detachment Registry, Shuichi Yamamoto, Takayuki Baba, Eiju Sato, Masayasu Kitahashi, Tomoaki Tatsumi, Gen Miura, Tomohiro Niizawa, Taiji Sakamoto, Keita Yamakiri, Toshifumi Yamashita, Hiroki Otsuka, Seiji Sameshima, Narimasa Yoshinaga, Shozo Sonoda, Akito Hirakata, Takashi Koto, Makoto Inoue, Kazunari Hirota, Yuji Itoh, Tadashi Orihara, Yoshinobu Emoto, Masahiko Sano, Hiroyuki Takahashi, Ryo Tokizawa, Hidetoshi Yamashita, Koichi Nishitsuka, Yutaka Kaneko, Katsuhiro Nishi, Akitoshi Yoshida, Shinji Ono, Hiroyuki Hirokawa, Kenji Sogawa, Tsuneaki Omae, Akihiro Ishibazawa, Shoji Kishi, Hideo Akiyama, Hidetaka Matsumoto, Ryo Mukai, Masahiro Morimoto, Mitsuru Nakazawa, Yukihiko Suzuki, Takashi Kudo, Kobu Adachi, Susumu Ishida, Kousuke Noda, Satoru Kase, Syouhei Mori, Ryo Ando, Michiyuki Saito, Tomohiro Suzuki, Kanji Takahashi, Yoshimi Nagai, Tadashi Nakauchi, Haruhiko Yamada, Shunji Kusaka, Daishi Tsujioka, Akitaka Tsujikawa, Kiyoshi Suzuma, Tatsuro Ishibashi, Koh-Hei Sonoda, Yasuhiro Ikeda, Riichiro Kohno, Keijiro Ishikawa, Mineo Kondo, Maki Kozawa, Takashi Kitaoka, Eiko Tsuiki, Yuichiro Ogura, Munenori Yoshida, Hiroshi Morita, Aki Kato, Yoshio Hirano, Kazuhiko Sugitani, Hiroko Terasaki, Takeshi Iwase, Yasuki Ito, Shinji Ueno, Hiroki Kaneko, Norie Nonobe, Taro Kominami, Noriyuki Azuma, Tadashi Yokoi, Hiroyuki Shimada, Hiroyuki Nakashizuka, Takayuki Hattori, Ari Shinojima, Yorihisa Kitagawa, Fumio Shiraga, Yuki Morizane, Shuhei Kimura, Tsunehiko Ikeda, Teruyo Kida, Takaki Sato, Masanori Fukumoto, Kazuyuki Emi, Hiroshi Nakashima, Masahito Ohji, Masashi Kakinoki, Osamu Sawada, Shinobu Takeuchi, Sumiyoshi Tanaka, Tomohiro Iida, Hideki Koizumi, Ichiro Maruko, Taiji Hasegawa, Akiko Kogure, Hiroyuki Iijima, Tomohiro Oshiro, Yasushi Tateno, Wataru Kikushima, Atsushi Sugiyama, Seigo Yoneyama, Kazuaki Kadonosono, Shimpei Sato, Shin Yamane

**Affiliations:** 1 Department of Ophthalmology, Kagoshima University Graduate School of Medical and Dental Sciences, Kagoshima, Japan; 2 The Japan-Retinal Detachment Registry Group, Osaka, Japan; 3 Department of Epidemiology and Preventive Medicine, Kagoshima University Graduate School of Medical and Dental Sciences, Kagoshima, Japan; 4 Department of Vision Informatics, Osaka University Graduate School of Medicine, Osaka, Japan; 5 Department of Ophthalmology, Chiba University, Chiba, Japan; 6 Department of Ophthalmology, Yamagata University, Yamagata, Japan; 7 Department of Ophthalmology, Kyorin Eye Center, Kyorin University School of Medicine, Tokyo, Japan; 8 Chiba University, Chiba, Japan; 9 Kagoshima University, Kagoshima, Japan; 10 Kyorin University, Tokyo, Japan; 11 Yamagata University, Yamagata, Japan; 12 Asahikawa Medical University Hospital, Asahikawa, Japan; 13 Gunma University, Maebashi, Japan; 14 Hirosaki University, Hirosaki, Japan; 15 Hokkaido University, Hokkaido, Japan; 16 Kansai Medical University Hospital, Osaka, Japan; 17 Kindai University Sakai Hospital, Osaka, Japan; 18 Kyoto University, Kyoto, Japan; 19 Kyushu University, Fukuoka, Japan; 20 Mie University, Mie, Japan; 21 Nagasaki University, Nagasaki, Japan; 22 Nagoya City University, Nagoya, Japan; 23 Nagoya University, Nagoya, Japan; 24 National Center for Child Health and Development, Tokyo, Japan; 25 Nihon University Hospital, Tokyo, Japan; 26 Okayama University, Okayama, Japan; 27 Osaka Medical School, Osaka, Japan; 28 Osaka Rosai Hospital, Osaka, Japan; 29 Shiga Medical University, Shiga, Japan; 30 Takeuchi Eye Clinic, Tokyo, Japan; 31 Tokyo Womens Medical College, Tokyo, Japan; 32 Yamanashi University, Yamanashi, Japan; 33 Yokohama City University Medical Center, Yokohama, Japan

**Keywords:** Medical research, Retinal diseases

## Abstract

The purpose of this study was to investigate the effects of surgeon-related factors on the surgical outcome of pars plana vitrectomy (PPV) and scleral buckling (SB) surgery on eyes with a rhegmatogenous retinal detachment (RRD). This was a nationwide, multicenter, observational study of the data in the Japan-RD Registry. Registered cases that had undergone surgery for a RRD by 128 accredited surgeons in 26 institutions were studied. The surgeon-related factors that significantly affected surgical success and visual outcomes of simple RRD treated by PPV or SB at 6 months postoperatively were analyzed and compared. Among 3446 registered cases, 2533 cases met the inclusion criteria with 1896 in the PPV group and 637 cases in the SB group. The median total number of lifetime cases was 150 and the rate of surgeries/year was 22. Multivariate regression analyses showed that the number and rate of surgeries/year were not significantly associated with the surgical outcome in the PPV group. However, surgeons with a higher average annual number of surgeries had significantly better surgical outcomes in the SB group (*P* = 0.038). Analyses of a nationwide registry showed that SB but not PPV surgeries require sufficient experience and case numbers to acquire and maintain skills to treat RRDs successfully.

## Introduction

The optimal method to treat a rhegmatogenous retinal detachment (RRD) has not been definitively determined. Currently, pars plana vitrectomy (PPV) with small-gauge instruments which is less invasive than the conventional 20-gauge instruments, is becoming the standard procedure, and it is preferred over scleral buckling (SB) surgery^1–4^. However, the pros and cons of these two methods are still being discussed.

Most of the earlier studies on the effectiveness of treatment for RRD have focused on factors related to the eye. Factors affecting the relationship between different surgical techniques and outcomes have also been examined^5–12^. However, the factors related to the skill and experience of the surgeons have not been examined in detail^13–18^. To provide the best treatment, it is important to know not only the best methods to treat the patient but also the surgeon-related factors.^13–20^.

There have been several studies on the effects of the experience of the surgeons on the outcome of ophthalmic surgery. For cataract surgery, a study of 38 surgeons showed that the complication rate decreased after a surgeon had performed ≥ 300 cataract surgeries^14^. For RRD surgery, Dugas et al. studied the findings of four surgeons and found that the less experienced surgeons required some time to achieve an acceptable success rates^21^. Mazinani et al. investigated the effects of learning on the success rate in eight doctors.^13^ They reported that the less experienced doctors had a higher learning effect, but the results could not be generalizable because of the small number of surgeons. To overcome this problem, it is necessary to analyze the performance of sufficient number of surgeons with varying degrees of experience in performing RRD surgery with standardized procedures.

Another challenge is determining the appropriate method to examine the data. Randomized clinical trials (RCT) are considered the standard for evaluating a specific therapy. However, RCT presents several methodological and practical difficulties^22,23^, but a multi-centered registry system should be helpful.

The Japanese Retina Vitreous Society (JRVS) has established a registry of patients who have undergone RRD to determine the optimal treatment of RRD^3,11,12,24^. The registry includes data from many ophthalmological surgeons and facilities and has a good mixture of experienced and less experienced surgeons performing standardized procedures which is better suited for our study.

The purpose of this study was to determine the effects of surgeon-related factors on the outcomes of PPV and SB surgery. To accomplish this, we analyzed the findings of registered cases treated by 128 accredited surgeons. The results of this study should provide basic data on the importance of surgeon-related factors on the outcomes of RRD surgery.

## Results

Of the 3446 eyes examined, 496 eyes were excluded because the RRD was caused by trauma, 172 eyes with a grade C proliferative vitreoretinopathy (PVR), 137 eyes with a choroidal detachment, 51 with a giant tear, 178 with a retinal break at the vitreous base, 95 with simple MH, and 36 with a retinal break of unknown cause. In addition, 241 eyes that had undergone combined PPV + SB surgery were excluded. Among these, there were 73 eyes (2.8%) with a simple RRD, 33 eyes with no information on the surgeon’s factors, and 3 eyes operated on by surgeon with less than 1 year of experience, and all were excluded. In the end, 913 eyes were excluded, and 2533 eyes (73.5%) were included in the statistical analyses. Of these, 1896 eyes (74.9%) had undergone PPV, and 637 eyes (25.1%) had undergone SB surgery.

**Figure 1:**
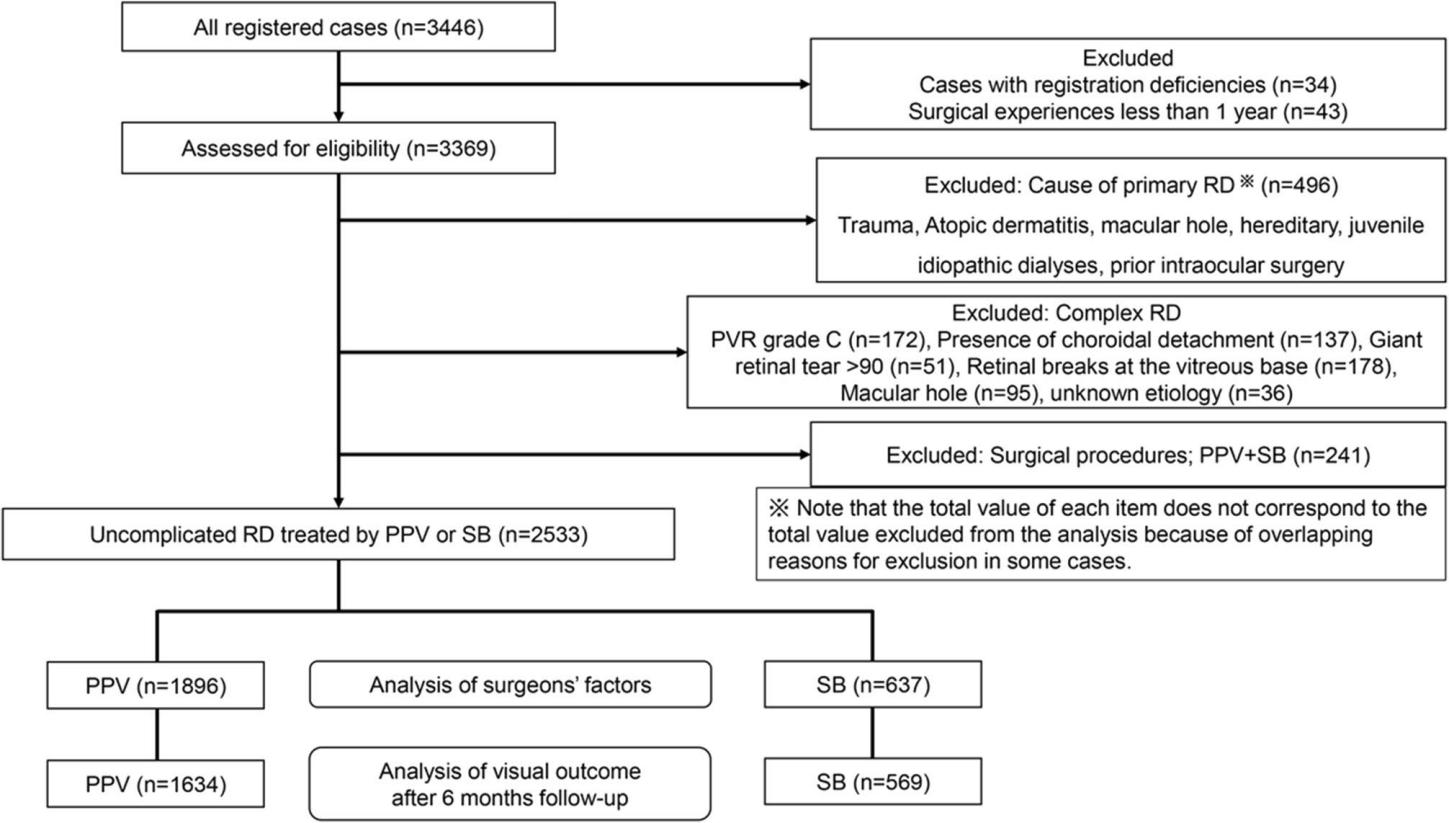
Flow-chart of present study progress. Because some cases had overlapping reasons for exclusion, the total number for each item does not equal the total number excluded from the analyses.

The changes in the visual acuity were examined in 2203 eyes of which 1634 eyes (74.2%) had undergone PPV and 569 eyes (25.8%) had undergone SB surgery Fig. 1.

### Analyses of surgeons

**Figure 2:**
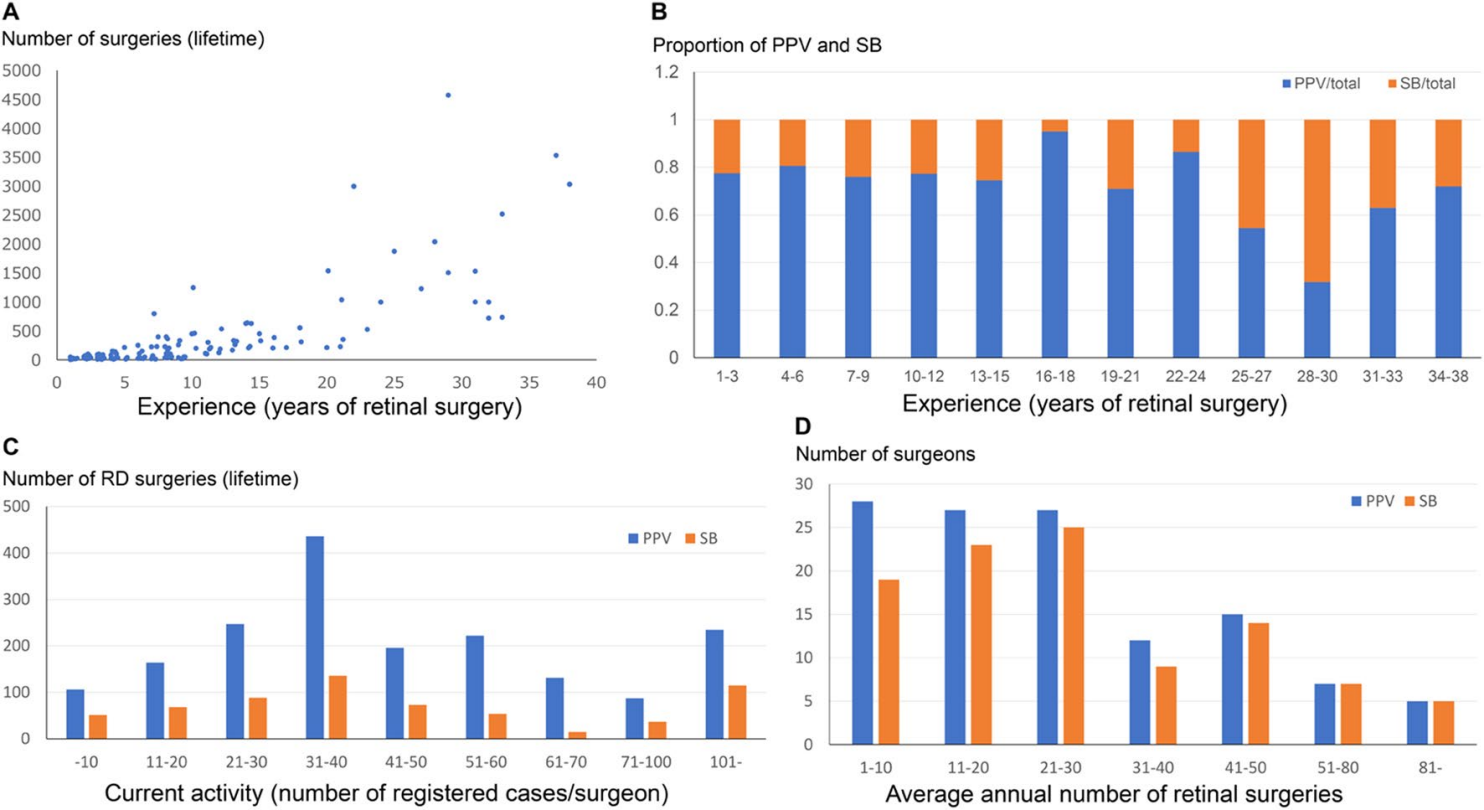
Breakdown of surgeons. (**A**) A scatter diagram of the surgeons and his/her lifetime experience of retinal surgeries. (**B**) Graph showing the proportion of PPV and SB cases performed relative to years of experience of retinal surgery. The proportion of SB surgeries increases with increasing years of experience with a peak at 28–30 years and then gradually decreases. (**C**) Number of surgical cases by current activity. Surgeons with current activity of 31–40 cases/surgeon performed the most retinal detachment surgeries by both PPV and SB surgery. (**D**) Number of surgeons by average number of annual retinal surgeries. For PPV, the number of surgeons decreased with an increase of the average annual number of retinal surgeries. While in SB, an average annual number of retinal surgeries had a peak of 21–30 cases/year.

The findings of 128 surgeons were studied. Their median years of experience was 8 years with a range from 1 to 38 years (Fig. 2A). The percentage of SB surgeries increased as the number of years of experience increased and peaked at 28–30 years. Thereafter, the percentage of SB surgeries decreased indicating that young surgeons preferred PPV to SB surgery (Fig. 2B).

The total number of RRD surgeries during the lifetime of each surgeon ranged from 10 to 4580 with a median of 150 cases. The total number of registered cases/surgeon during the study period ranged from 1 to 144 cases with a median of 21 cases, and many of the RRD surgeries were performed by surgeons registering 31 to 40 cases during the period of both PPV and SB surgeries (Fig. 2C).

### Average number of annual retinal surgery

For PPV, the number of surgeons decreased as the annual number of retinal surgeries increased. For SB, the average annual number of retinal surgeries had a peak of 21–30 cases/year (Fig. 2D).

The factors that were related to the surgical success were determined. For eyes that had undergone PPV, the results showed that the factors significantly associated with poor outcome of the surgery included prior surgery of the patient (*P* = 0.036, Chi-square test), poor preoperative BCVA *(P* = 0.007, Mann–Whitney-U test), location of the retinal break *(P* < 0.001 Chi-square test), presence of PVR-B (*P* = 0.001, Chi-square test), and intraoperative complications *(P* = 0.004, Chi-square test; Supplementary Table S1).

For the eyes that had undergone SB surgery, only poor preoperative BCVA *(P* < 0.001, Mann–Whitney-U test) was significantly associated with poor postoperative outcome (Supplementary Table S2).

### Surgeon-related factors and surgical outcomes

In the PPV group, the sex, age, prior surgery, preoperative BCVA, location of the retinal break, PVR, and intraoperative complications were used as covariates in the logistic regression analyses. In the SB group, the sex, age, and preoperative visual acuity were used as covariates. The results of the risk of failure by the surgeon-related factors in eyes that underwent PPV are shown in Table 1. The risk of failure increased as the number of surgeries increased (*P* for trend = 0.056). Similarly, the risk of failure was higher for surgeons who had more years of experience compared to the group with fewer years of experience (Q1). This was significantly higher in Q2 and Q4. The surgeons with current activity had the lowest risk of failure in the group with the highest number of registered cases (Q4) at 0.63 (95% CI: 0.36, 1.11). However, this was not significant.

**Table 1:** Relationship between factors associated with surgeons’ experience and surgical outcomes in PPV cases.

Variable	No. of eyes(%)			
	Success	Failure	*P* value*	aOR (95%CI)**
**Total number of surgeries by quartile (range)**				
Q1 (4–90)	470 (94.6)	27 (5.4)	0.325	reference
Q2 (91–229)	435 (94.0)	28 (6.0)		1.03 (0.59, 1.79)
Q3 (230–460)	446 (93.7)	30 (6.3)		1.16 (0.67, 2.00)
Q4 (527–4574)	422 (91.7)	38 (8.3)		1.64 (0.97, 2.77)
Median (range)	226 (0–4,574)	253 (0–3,534)	0.108	*P* for trend = 0.056
**Duration of surgical experiences into quartile (range)**				
Q1 (1–3)	466 (95.7)	21 (4.3)	0.066	reference
Q2 (4–8)	509 (92.2)	43 (7.8)		1.92 (1.12, 3.32)
Q3 (9–14)	378 (94.2)	23 (5.7)		1.41 (0.76, 2.61)
Q4 (15–38)	420 (92.1)	36 (7.9)		1.82 (1.04, 3.20)
Median (range)	8 (1–38)	8 (1–38)	0.028	*P* for trend = 0.130
**Experience: Number of cases/year by quartile (range)**				
Q1 (2.1–20.1)	445 (93.5)	31 (6.5)	0.763	reference
Q2 (20.3–30)	470 (94.4)	28 (5.6)		0.88 (0.51, 1.50)
Q3 (30.2–45.1)	428 (93.4)	30 (6.6)		1.06 (0.62, 1.80)
Q4 (45.4–157.7)	430 (92.7)	34 (7.3)		1.36 (0.82, 2.34)
Median (range)	30 (0–158)	31 (0–136)	0.630	P for trend = 0.183
**Activity: Number of registered cases by quartile (range)**				
Q1 (1–28)	449 (92.6)	36 (7.4)	0.09	reference
Q2 (30–40)	442 (94.4)	26 (5.6)		0.71 (0.42, 1.21)
Q3 (41–63)	446 (91.8)	40 (8.2)		1.06 (0.66, 1.71)
Q4 (65–144)	436 (95.4)	21 (4.6)		0.63 (0.36, 1.11)
Median (range)	40 (1–144)	40 (2–122)	0.274	P for trend = 0.330

*Chi-square test or Mann–Whitney U test.

**Adjusted odds ratio (aOR) based on multivariate logistic regression analysis. This model was adjusted for sex, age, best-corrected visual acuity, location of the largest tears or holes, presence of proliferative vitereoretinopathy (PVR; B), and intraoperative complications.

PPV, pars plana vitrectomy; aOR, adjusted odds ratio; CI, confidence interval.

In SB surgery, there was a lower risk of failure (higher chance of success) with increasing number of cases (*P* = 0.220 in Model 1) and years of experience (*P* = 0.438 in Model 1). However, the differences were not significant. The risk of failure was significantly lower for the surgeons with the highest number of retinal surgical cases/year (OR: 0.31, 95% CI: 0.11, 0.86; *P* for trend = 0.038, Model 1 in Table 2). Because of the small number of patients who had SB, we limit the number of covariates to the basic variables of sex, age, and best-corrected visual acuity that had been shown to be statistically significant in the univariate analyses (Table S2) in the statistical model (Model 1). In the sensitivity analysis using the same statistical model with PPV, similar results were obtained (Model 2).

**Table 2:** Relationship in SB cases between factors associated with surgeons’ experience and surgical outcomes.

Variable	No. of eyes(%)		*P* value*	aOR (95%CI)**	
	Success	Failure		Model 1	Model 2
**Total number of years of surgical experiences into quartile (range)**					
Q1 (10–59)	151 (91.0)	15 (9.0)	0.560	reference	reference
Q2 (69–229)	145 (92.9)	11 (7.1)		0.74 (0.32, 1.70)	0.74 (0.32, 1.72)
Q3 (230–1,002)	149 (94.9)	8(5.1)		0.66 (0.26, 1.64)	0.63 (0.25, 1.60)
Q4 (1,040–4,574)	148(93.7)	10 (6.3)		0.59 (0.25, 1.41)	0.61 (0.25, 1.46)
Median (range)	230 (0, 4,574)	139 (14, 4,574)	0.126	P for trend = 0.220	P for trend = 0.233
**Duration of surgical experiences into quartile (range)**					
Q1 (1–4)	155 (93.4)	11 (6.6)	0.870	reference	reference
Q2 (5–9)	142 (91.6)	13 (8.4)		1.05 (0.43, 2.54)	1.07 (0.44, 2.61)
Q3 (10–21)	148 (88.1)	10 (11.9)		0.84 (0.34, 2.08)	0.83 (0.33, 2.06)
Q4 (22–38)	148 (93.7)	10 (6.3)		0.74 (0.29, 1.89)	0.76 (0.30, 1.96)
Median (range)	9 (1–38)	8 (1–33)	0.908	P for trend = 0.438	P for trend = 0.456
**Rate of success: Number of cases per year into quartile (range)**					
Q1 (0–15.8)	141 (89.2)	17 (10.8)	0.107	reference	reference
Q2 (16.6–30)	147 (93.6)	10 (6.4)		0.71 (0.30, 1.68)	0.70 (0.29, 1.67)
Q3 (30.2–49.5)	153 (93.3)	11 (6.7)		0.81 (0.35, 1.86)	0.78 (0.33, 1.84)
Q4 (50–157.7)	152 (96.2)	6 (3.8)		0.31 (0.11, 0.86)	0.32 (0.12, 0.87)
Median (range)	30 (2–158)	21 (2–158)	0.02	P for trend = 0.038	P for trend = 0.040
**Experience: Number of registered cases into quartile (range)**					
Q1 (1–25)	153 (90.0)	17 (10.0)	0.179	reference	reference
Q2 (26–39)	150 (96.2)	6 (3.8)		0.35 (0.13, 0.93)	0.35 (0.13, 0.96)
Q3 (40–62)	148 (93.7)	10 (6.3)		0.53 (0.23, 1.24)	0.53 (0.23, 1.23)
Q4 (65–144)	142 (92.8)	11 (7.2)		0.56 (0.24, 1.30)	0.57 (0.24, 1.34)
Median (range)	39 (1–144)	39 (2–144)	0.160	P for trend = 0.224	P for trend = 0.242

SB, scleral buckling; aOR, adjusted odds ratio; CI, confidence interval.

*Chi-square test or Mann–Whitney U test.

**Adjusted odds ratio (aOR) based on multivariate logistic regression analysis. Model 1 was adjusted for sex, age, and best-corrected visual acuity. Model 2 was the same model used in PPV cases; adjusted for sex, age, best-corrected visual acuity, location of the largest tears or holes, presence of proliferative vitereoretinopathy (PVR; B), and intraoperative complications.

### Best-corrected visual acuity (BCVA)

The effects of the surgeries on the BCVA were studied in 2,203 eyes with 1,634 undergoing PPV and 569 undergoing SB surgery. At 6 months postoperatively, 786 PPV patients (48.1%) and 128 SB patients (22.5%) had a significant improvement of their BCVA (Supplementary Table S3). The effects of the baseline characteristics on the visual acuity at the 6 months after the surgery are presented in Supplementary Tables S4 and S5. In both the PPV and SB groups, the preoperative visual acuity (*P* < 0.001 for both group) and the presence of macular detachment (*P* < 0.001 for both group) were factors significantly associated with an improvement of the visual acuity. For the PPV group, the sex (*P* = 0.037), size of the largest break (*P* = 0.024), PVR stage (*P* = 0.017), surgical time (*P* = 0.007), with or without drainage retinotomy (*P* = 0.010), and the presence of intraoperative complications (*P* = 0.035) were significantly associated with the visual outcome (Supplementary Table S4). For the SB group, the patients’ age (*P* = 0.007), prior ocular surgery (*P* = 0.015), and lens status (*P* = 0.002) were significantly associated with the visual outcome (Supplementary Table S5). Supplementary Tables S6 and S7 show the effects of the surgeon’s experience and activities on the visual acuity at 6 months after the surgery. For the PPV group, there was a negative association between the number of total surgical experience and worsened visual outcome (*P* for trend = 0.024, Supplementary Table S6). For the SB groups, the trend was not significant for any of the factors (Supplementary Tables S7).

## Discussion

The background of the RD patients and surgical outcomes were thoroughly studied and published using same registry data^3,11,12,24,29^. Unlike these reports, the purpose of this study was to determine the surgeon-related factors associated with the surgical outcomes of RRD surgery. The results showed that the average number of retinal surgeries per year was significantly associated with the surgical success/failure ratio in eyes that had undergone SB surgery but not PPV surgery. To the best of our knowledge, this type of analyses of a nationwide registry data has not been published and so they are new findings.

It is generally assumed that the skills and experience of the surgeon play a major role in the outcomes of surgical treatments, however there are other factors that need to be considered, e.g., the type of treatment selected by the surgeon^4,19,22,23,25,26^. Therefore, we took two steps to minimize the bias caused by the preoperative characteristics of the patients. The first step was to confine cases to those with relatively simple RRD and limit the type of surgeries to either SB or PPV. Thus, cases treated by combined PPV + SB, which is usually performed on complicated or severe RRD, were excluded^11,12^. The second step was to use a multivariate model of statistical analyses so that the effects of potential confounders could be tested. With these two steps, we believe that reasonable comparisons of the surgeons-related factors could be made with greater validity. One may argue that PPV + SB surgery had the best surgical results for pseudophakic eyes and should be included.^28^ However, this was not a study to compare the superiority of PPV to SB or vice versa. In addition, PPV + SB was performed on only 2.8% for simple RRD in this registry. Our findings showed that the exclusion of PPV + SB did not appear to cause any strong bias.

The total number of lifetime RRD surgeries and years of experience were not significant factors for each type of surgery. After the surgical numbers attained a plateau, the results did not improve with additional number of cases and years of experience^21^. This ceiling effect might have obscured the effects of the above factors in this study.

Next, we examined whether the average annual number of retinal surgeries was significantly associated with the outcomes. The results showed that there was a significant trend that the surgeons with a higher number of retinal surgeries per year were associated with better surgical results in SB surgery. This indicated that a certain number of cases per year is required to maintain a good skill level. For SB surgeries, it may be necessary to perform SB surgeries continuously to maintain the skills. The SB surgical procedures vary considerably and requires careful selection of the patient, type of buckle, and buckle orientation to treat the RRD successfully. These limitations would require constant performance. Thus, it is reasonable that surgeons who perform a certain number of SB operations/year have a higher success rate.

On the other hand, the difference of the anatomical outcomes was not observed in the PPV cases. There was a non-significant trend for a lower anatomical success rate in surgeons with higher numbers of PPV while there was a negative association between the number of total surgical experience and worsened visual outcome. This is difficult to interpret. We adjusted for factors unrelated to the surgeons that affected surgical outcomes, so it was unlikely due to a case selection bias. A possible explanation was that even inexperienced surgeons can achieve results comparable to those of experienced surgeons in PPV for eyes with simple RRD. The learning curve may be steeper for PPV than SB to reach a certain level of success.

A good visual acuity is the most important endpoint in any ocular surgery, but there was no significant association between the surgeon-related factors and the visual acuity. Because the postoperative visual acuity is strongly influenced by the preoperative visual acuity, it may be strong enough to mask the surgeon-related factors^9^. This indicated that it is not appropriate to use the visual acuity alone to evaluate the outcome of RRD surgery.

A recent large study in the USA reported that SB was superior to PPV in visual outcomes and surgical success for phakic RRD^27,28^. Our earlier studies also showed that SB was superior to PPV in simple macula-on RRD^11,29^. Even though the success rate of PPV was equal to that of SB in other studies, PPV has risks of late complications^9,10,23,30^. So, SB surgery should be chosen more than PPV. However, SB is not frequently chosen in the real world especially by young and inexperienced surgeons as was found in this study^1–3^. Importantly we found that PPV achieved an acceptable level of success even by inexperienced surgeons. Thus, PPV has the advantage over SB in that the surgeons without much experience can achieve good outcomes. For this reason, young surgeons may choose PPV over SB. Furthermore, the fact that a certain level of experience is continuously required to maintain the skills for SB surgeries may be the reason why surgeons are reluctant to choose SB. If the outcome of SB is better, it might be better to select SB more from the point of view of the patient, and it may be necessary to develop an educational system or health policies that encourages the choice of SB.

There are limitations in this study. First, this was a retrospective study with its inherent biases. Although we did adjust for the preoperative and surgeon-nonrelated factors, there might be unknown factors that were not adjusted for. To lessen this problem, a prospective RCT study is necessary. But it is practically difficult to perform a RCT for already established treatments and a registry method such as the present one seems to be the only realistic method^22,23^.

Second, this was a cross-sectional study and did not show the longitudinal changes of the surgeons’ skills. Because there was a trend that experienced surgeons chose SB more than young and inexperienced surgeons, it could be possible that the factor of experience was more evident in SB than PPV. Nonetheless, because the significance was found only in the annual number of surgeries/year, the amount of experience would not be large. Most notably, this is not a study to compare PPV and SB. This study shows the surgical results for simple RRD by the same surgeons during the same period. To compare the surgeon-related factor of PPV and SB, another study of more than 100 surgeons may be necessary, but it would be difficult practically. Finally, the results cannot be generalized to all regions of the world because the surgical procedures are affected by the educational system, the insurance system, and customs of each region.

In conclusion, a higher success rate was associated with surgeons with higher average number of SB surgeries/year. This finding gives us insight on the importance of lifelong education starting from the time of residency training. To realize the beneficial treatment for patients in the future, the surgeon-related data needs to be verified for ophthalmic surgery, and this is an important step in that direction.

## Methods

### Ethics statement

The protocol of this study was approved by the Ethics Committee of the Kagoshima University (#140093, 28-38), and all other institutions^11,12,24,29^. The study procedures conformed to the tenets of the Declaration of Helsinki and the New Clinical Research Act of Japan. This study was an observational study based on the information collected as a result of standard care and identifiable information was de-identified in this registry study, the requirement for individual written informed consent from the patients was approved to be waived by all the hospitals or institutes except for the Kyushu University hospital Ethics Committee. For the participants of Kyushu University, a written informed consent was obtained from all of the participants.

### Data registry

The Japan-Retinal Detachment (J-RD) registry was created for 26 ophthalmological centers throughout Japan^11,12,24,29^. All of the institutions were members of the JRVS, and all surgeons were accredited by the Japanese Ophthalmological Society. The results of all of the RRD cases treated in each facility from February 2016 to March 2017 were registered. More than 50 items/eye were entered including the preoperative findings, surgical details, and postoperative findings. The details of the protocols have been published^11,12,24,29^.

### Definition of RRD

In our earlier study, complicated RRDs were found to have significantly poorer prognosis^11,12,24^. So, 496 eyes with complicated RRD were excluded. In addition, eyes with a proliferative vitreoretinopathy (PVR) classification of C or higher, choroidal detachment, giant tears, a retinal break near the vitreous base, and unknown breaks which were refractory RRD were also excluded^7,11,12^. Based on reports of the selection procedures, cases treated by a combination of PPV + SB were excluded because they were selected for more difficult cases and the findings may confound the surgeon-related factors^3,11,12,24^. Nevertheless, they made up only 2.8% of the simple RRD cases. These exclusions were determined by the JRVS analysis committee at the planning stage. Therefore, the studied cases can be classified as simple RRD cases (Fig. 1). The surgical methods and follow-up examinations were determined by the surgeon and the follow-up was done in a standard manner as published^11,12,24^.

### Definition of surgical success and failure

Eyes with a RRD were considered “successfully treated” if the retina was attached at 6 months after a single surgery with no tamponade. Cases that underwent reoperation for reasons other than planned silicone removal were regarded as surgically unsuccessful. Thus, all cases other than surgically success were classified as surgical failures.

### Definition of improvement of visual acuity

The visual outcomes were determined for each patient at 6 months after the surgery. Eyes with visual acuity of 0.2 logarithm of the minimum angle of resolution (logMAR) units or better than the preoperative visual acuity or those with visual acuity of 20/20 or better at 6 months were classified as “an improvement of the visual acuity”. Those without an improvement of the visual acuity, were classified as a lack of improvement of the visual acuity.

### Surgeon-nonrelated factors affecting surgical success

Our earlier studies showed that the surgical outcomes differed depending on the nonrelated surgeon or preoperative factors, e.g., location of the retinal break, presence of macular detachment, and other factors even for simple RRD of the same degree^11,12^. Therefore, we also analyzed factors related to the surgical success such as the age, sex, type of retinal break, and other factors which were determined by the JRVS committee preoperatively (Supplemental Tables S1 and S2).

### Definition of surgeon-related factors

The surgeon-related factors that could be expressed in objective values were selected by the J-RD registry committee. The number of years of experience in retinal surgery and the number of retinal surgeries performed during the lifetime of the surgeon were classified as the experience of the surgeons. The number of registered cases during the current period was selected as the current activity of the surgeon. In addition, the annual average number of surgeries was calculated by dividing the total number of surgeries performed by the number of years of experience to determine the continuity of surgeries, i.e., number of cases/years. Surgeons with incomplete registration, e.g., omission of information, were excluded.

### Statistical analyses

The primary outcome measure was the surgical results, success/failure at 6 months postoperatively, and the secondary outcome was the improvement in the visual acuity at 6 months postoperatively. Both were analyzed separately for the SB and PPV groups. Chi-square tests and Mann–Whitney U tests were used to determine the significance of the differences between the cases with nominal and continuous variables between each outcome group. The association between surgical success or visual acuity improvements as the objective variable and surgeon-related factors were analyzed by multivariate logistic regression model. The results were divided into quartiles by the value of each surgeon’s factor, number of lifetime vitreoretinal surgery cases, years of experience, cases/years, and number of registered cases. The values below the first quartile (Q1) were designated as the reference values, and the odds ratios and 95% confidence intervals were estimated for the risk of failure or no improvement in the vision at 6 months in the other groups. The likelihood ratio test was used to determine the *P* values for the trends. The STATA16 was used for the statistical analyses, and a two-tailed test was performed with the significance level set at 0.05.

## Supplementary Information

Supplementary Information 1.

Supplementary Information 2.

Supplementary Information 3.

Supplementary Information 4.

Supplementary Information 5.

Supplementary Information 6.

Supplementary Information 7.

## References

[1] Eibenberger K, Georgopoulos M, Rezar-Dreindl S, Schmidt-Erfurth U, Sacu S (2018). Development of surgical management in primary rhegmatogenous retinal detachment treatment from 2009 to 2015. Curr. Eye Res..

[2] Cho, G. E., Kim, S. W., Kang, S. W.; Korean Retina Society. Changing trends in surgery for retinal detachment in Korea. *Korean J. Ophthalmol.* **28**, 451–459 (2014). 10.3341/kjo.2014.28.6.451 PMC4239463 25435747

[3] Nishitsuka K (2020). Preoperative factors to select vitrectomy or scleral buckling for retinal detachment in microincision vitrectomy era. Graefes. Arch. Clin. Exp. Ophthalmol..

[4] Keller J, Haynes RJ, Sparrow JM (2016). Sequential hypothesis testing to characterise the learning curve and monitor surgical performance in retinal detachment surgery. Ophthalmologica.

[5] Heimann H (2006). Primary vitrectomy for rhegmatogenous retinal detachment: An analysis of 512 cases. Graefes. Arch. Clin. Exp. Ophthalmol..

[6] Adelman, R. A., Parnes, A. J., Ducournau, D.; European Vitreo-Retinal Society (EVRS) Retinal Detachment Study Group. Strategy for the management of uncomplicated retinal detachments: The European vitreo-retinal society retinal detachment study report 1. *Ophthalmology* **120**, 1804–1808 (2013). 10.1016/j.ophtha.2013.01.070 23601799

[7] Adelman, R. A., Parnes, A. J., Sipperley, J. O., Ducournau, D.; European Vitreo-Retinal Society (EVRS) Retinal Detachment Study Group. Strategy for the management of complex retinal detachments: The European vitreo-retinal society retinal detachment study report 2. *Ophthalmology* **120**, 1809–1813 (2013). 10.1016/j.ophtha.2013.01.056 23601805

[8] Yoon, Y. H., Shwu-Jiuan, S. & Terasaki, H. Primary vitrectomy in rhegmatogenous retinal detachment. In *Retina* (ed. Ryan, S. J.). 1712–1720 (CV Mosby, St. Louis, 2012)

[9] Znaor L, et al. Pars plana vitrectomy versus scleral buckling for repairing simple rhegmatogenous retinal detachments. *Cochrane Database Syst. Rev.* **3**, CD009562 (2019). 10.1002/14651858.CD009562.pub2 PMC6407688 30848830

[10] Haugstad M, Moosmayer S, Bragadόttir R (2017). Primary rhegmatogenous retinal detachment—surgical methods and anatomical outcome. Acta Ophthalmol..

[11] Baba T (2021). Visual outcomes after surgery for primary rhegmatogenous retinal detachment in era of microincision vitrectomy: Japan-Retinal Detachment Registry Report IV. Br. J. Ophthalmol..

[12] Koto T (2021). Six-months primary success rate for retinal detachment between vitrectomy. Retina.

[13] Mazinani BA, Rajendram A, Walter P, Roessler GF (2012). Does surgical experience have an effect on the success of retinal detachment surgery?. Retina.

[14] Gupta S, Haripriya A, Vardhan SA, Ravilla T, Ravindran RD (2018). Residents' learning curve for manual small-incision cataract surgery at Aravind Eye Hospital, India. Ophthalmology.

[15] Lee JJ (2017). Learning curve for endoscopic endonasal dacryocystorhinostomy. Korean J. Ophthalmol..

[16] Eppsteiner RW, Csikesz NG, McPhee JT, Tseng JF, Shah SA (2009). Surgeon volume impacts hospital mortality for pancreatic resection. Ann. Surg..

[17] Miki D, Hida T, Hotta K, Shinoda K, Hirakata A (2001). Comparison of scleral buckling and vitrectomy for retinal detachment resulting from flap tears in superior quadrants. Jpn. J. Ophthalmol..

[18] Emami-Naeini P (2019). Pneumatic retinopexy experience and outcomes of vitreoretinal fellows in the United States: A multicenter study. Ophthalmol. Retina..

[19] Katlic MR, Coleman J, Russell MM (2019). Assessing the performance of aging surgeons. JAMA.

[20] Grodin MH, Johnson TM, Acree JL, Glaser BM (2008). Ophthalmic surgical training: A curriculum to enhance surgical simulation. Retina.

[21] Dugas B (2009). The learning curve for primary vitrectomy without scleral buckling for pseudophakic retinal detachment. Graefes. Arch. Clin. Exp. Ophthalmol..

[22] Lonjon G (2014). Comparison of treatment effect estimates from prospective nonrandomized studies with propensity score analysis and randomized controlled trials of surgical procedures. Ann. Surg..

[23] Farrokhyar F (2010). Randomized controlled trials of surgical interventions. Ann Surg..

[24] Sakamoto T (2020). Japan-Retinal Detachment Registry Report I: Preoperative findings in eyes with primary retinal detachment. Jpn J Ophthalmol..

[25] Prystowsky JB, Bordage G, Feinglass JM (2002). Patient outcomes for segmental colon resection according to surgeon's training, certification, and experience. Surgery.

[26] Sagong M, Chang W (2010). Learning curve of the scleral buckling operation: lessons from the first 97 cases. Ophthalmologica.

[27] Ryan EH (2020). Primary retinal detachment outcomes study report number 2: Phakic retinal detachment outcomes. Ophthalmology.

[28] Joseph DP (2020). Primary retinal detachment outcomes study: Pseudophakic retinal detachment outcomes: Primary retinal detachment outcomes study report number 3. Ophthalmology.

[29] Kawano, S., Imai, T., Sakamoto, T.; Japan-Retinal Detachment Registry Group. Scleral buckling versus pars plana vitrectomy in simple phakic macula-on retinal detachment: a propensity score-matched, registry-based study [published online ahead of print, 2021 Jan 29]. *Br .J. Ophthalmol.*10.1136/bjophthalmol-2020-318451 (2021). 10.1136/bjophthalmol-2020-318451 33514527

[30] Chang S (2006). LXII Edward Jackson lecture: open angle glaucoma after vitrectomy. Am. J. Ophthalmol..

