# Supplementary material for: Effect of surgeon-related factors on outcome of retinal detachment surgery: analyses of data in Japan-retinal detachment registry
Source: Sci Rep. 2022 Mar 10;12:4213. doi: 10.1038/s41598-022-07838-5 (PMC8913601; doi:10.1038/s41598-022-07838-5)
Supplement: Supplementary file 1 — Supplementary Information 1. [file 41598_2022_7838_MOESM1_ESM.docx]

**Effect of Surgeon-Related Factors on Outcome of**

**Retinal Detachment Surgery: Analyses of Data**

**in Japan-Retinal Detachment Registry**

Keita Yamakiri^1,2^, Taiji Sakamoto^1,2^, Chihaya Koriyama^3^, Ryo Kawasaki ^2,4^, Takayuki Baba ^2,5^, Koichi Nishitsuka ^2,6^, Takashi Koto ^2,7^, Hiroto Terasaki ^1^ on behalf of Japan Retinal Detachment Registry

^1^Department of Ophthalmology, Kagoshima University Graduate School of Medical and Dental Sciences; ^2^The Japan-Retinal Detachment Registry Group; ^3^ Department of Epidemiology and Preventive Medicine, Kagoshima University Graduate School of Medical and Dental Sciences;^4^Department of Vision Informatics, Osaka University Graduate School of Medicine; ^5^Department of Ophthalmology, Chiba University; ^6^Department of Ophthalmology, Yamagata University; and ^7^Department of Ophthalmology, Kyorin Eye Center, Kyorin University School of Medicine.

| **Table S1. Baseline characteristics and success and failure rates at 6 months of cases that underwent pars plana vitrectomy (PPV).** (Online only) | | | |
| --- | --- | --- | --- |
| **Characteristics** | **No. of eyes (%)** | | ***P* value*** |
|  | Success | failure |  |
| **Sex** | | | |
| All | 1773 (93.5) | 123 (6.5) | 0.381 |
| Male | 1186 (93.1) | 87 (6.9) |  |
| **Age (years)** | | | |
| <50 | 280 (94.0) | 18 (6.0) | 0.144 |
| 50- | 593 (93.4) | 42 (6.6) |  |
| 60- | 612 (94.6) | 35 (5.4) |  |
| 70- | 288 (90.9) | 29 (9.1) |  |
| Median (range) | 60 (12, 96) | 61 (30, 85) | 0.148 |
| **Cause of retinal detachment** | | | |
| Retinal tear related to traction | 1,591 (94.0) | 107 (6.0) | 0.494 |
| Retinal hole, atrophic hole or retinal atrophy with lattice degeneration | 138 (92.7) | 11 (7.3) |  |
| Others | 44 (89.8) | 5 (10.2) |  |
| **Status of macula** | | | |
| Macula on | 788 (92.5) | 64 (7.5) | 0.176 |
| Macula off | 970 (94.3) | 59 (5.7) |  |
| Unknown | 15(100) | 0(0) |  |
| **Previous ocular surgery** | | | |
| Yes | 389 (91.3) | 37 (8.7) | 0.036 |
| No | 1,384 (94.1) | 86 (5.9) |  |
| **Best-corrected visual acuity into quartile (range)**** | | | |
| Q1 (-0.30, -0.08) | 452 (94.4) | 27 (5.6) | 0.027 |
| Q2 (0, 0.10) | 362 (95.8) | 16 (4.2) |  |
| Q3 (0.15, 0.82) | 481 (93.6) | 33 (6.4) |  |
| Q4 (0.83, 4.0) | 475 (91.0) | 47 (9.0) |  |
| Median (range) | 0.15 (-0.3) 4.0) | 0.40 (-0.18, 3.0) | 0.007 |
| **Lens status** | | | |
| Phakic | 1,458 (93.9) | 96 (6.1) | 0.380 |
| Aphakic | 7 (87.5) | 1 (12.5) |  |
| Pseudophakic eye | 314 (92.1) | 27 (7.9) |  |
| **Location of largest break** | | | |
| Superior | 1,402 (94.7) | 79(5.3) | <0.001 |
| Inferior/posterior pole | 371 (89.4) | 44 (10.6) |  |
| **Size of largest break (degrees)** | | | |
| 0-30 | 1,622 (93.9) | 106 (6.1) | 0.132 |
| 30-60 | 131 (89.7) | 15 (10.3) |  |
| 60-90 | 20 (90.9) | 2 (9.1) |  |
| **Type of break** | | | |
| Hole | 154 (92.8) | 12 (7.2) | 0.714 |
| Tear | 1,619 (93.5) | 111 (6.5) |  |
| **PVR** | | | |
| PVR stage B | 81 (85.3) | 14 (14.7) | 0.001 |
| PVR stage N | 1,692 (93.9) | 109 (6.1) |  |
| **Surgical time into quartile (range)** | | | |
| Q1 (10, 51) | 479 (95.4) | 23 (4.6) | 0.059 |
| Q2 (52, 70) | 471 (92.7) | 37 (7.3) |  |
| Q3 (71, 97) | 434 (94.3) | 26 (5.7) |  |
| Q4 (98, 372) | 389 (91.3) | 37 (8.7) |  |
| Median(range) | 68 (10, 300) | 72 (22, 216) | 0.008 |
| **Drainage retinotomy** | | | |
| Performed | 497 (92.2) | 42 (7.8) | 0.337 |
| Not performed | 1,275 (94.0) | 81 (6.0) |  |
| Unknown | 1(100) | 0(0) |  |
| **Intraoperative adjuvant use** | | | |
| Yes | 1,587 (93.8) | 105 (6.2) | 0.152 |
| No | 186 (10.5) | 18 (14.5) |  |
| **Intraoperative complications** | | | |
| Yes | 249 (89.6) | 29 (10.4) | 0.004 |
| No | 1,524 (94.2) | 94 (5.8) |  |

PPV, pars plana vitrectomy; stage N, retinal detachment with stage A proliferative vitreoretinopathy (PVR) and no PVR

**P* values for categorical and continuous variables obtained by chi-square test and Mann-Whitney U test, respectively.

**Decimal values were converted to the logarithm of the minimal angle of resolution (logMAR) units.
