# Supplementary material for: Effect of surgeon-related factors on outcome of retinal detachment surgery: analyses of data in Japan-retinal detachment registry
Source: Sci Rep. 2022 Mar 10;12:4213. doi: 10.1038/s41598-022-07838-5 (PMC8913601; doi:10.1038/s41598-022-07838-5)
Supplement: Supplementary file 2 — Supplementary Information 2. [file 41598_2022_7838_MOESM2_ESM.docx]

**Effect of Surgeon-Related Factors on Outcome of**

**Retinal Detachment Surgery: Analyses of Data**

**in Japan-Retinal Detachment Registry**

Keita Yamakiri^1,2^, Taiji Sakamoto^1,2^, Chihaya Koriyama^3^, Ryo Kawasaki ^2,4^, Takayuki Baba ^2,5^, Koichi Nishitsuka ^2,6^, Takashi Koto ^2,7^, Hiroto Terasaki ^1^ on behalf of Japan Retinal Detachment Registry

^1^Department of Ophthalmology, Kagoshima University Graduate School of Medical and Dental Sciences; ^2^The Japan-Retinal Detachment Registry Group; ^3^ Department of Epidemiology and Preventive Medicine, Kagoshima University Graduate School of Medical and Dental Sciences;^4^Department of Vision Informatics, Osaka University Graduate School of Medicine; ^5^Department of Ophthalmology, Chiba University; ^6^Department of Ophthalmology, Yamagata University; and ^7^Department of Ophthalmology, Kyorin Eye Center, Kyorin University School of Medicine.

| **Table S2. Baseline characteristics and success or failure rates at 6 months in cases that underwent scleral buckling.** (Online only) | | | |
| --- | --- | --- | --- |
| **Characteristics** | **No. of eyes (%)** | | **P value*** |
|  | Success | Failure |  |
| **Sex** | | | |
| All | 596(93.1) | 44(6.9) |  |
| Male | 359 (92.3) | 30 (7.7) | 0.316 |
| **Age (years)** | | | |
| <50 | 379 (92.7) | 30 (7.3) | 0.082 |
| 50- | 123 (95.3) | 6 (4.6) |  |
| 60- | 67 (95.7) | 3 (4.3) |  |
| 70- | 24 (82.8) | 5 (17.2) |  |
| Median (range) | 42 (11, 94) | 46 (14, 80) | 0.431 |
| **Cause of retinal detachment** | | | |
| Retinal tears related to traction | 247 (91.1) | 24 (8.9) | 0.103 |
| Retinal holes, atrophic holes, or retinal atrophy with lattice degeneration | 334 (94.9) | 18 (5.1) |  |
| Others | 12 (85.7) | 2 (14.3) |  |
| **Status of macula** | | | |
| Macula on | 195 (89.9) | 22 (10.1) | 0.065 |
| Macula off | 395 (94.7) | 22 (5.3) |  |
| Unknown | 3 (100) | 0 (0) |  |
| **Previous ocular surgery** | | | |
| Yes | 60 (93.8) | 4 (6.2) | 0.827 |
| No | 533 (93.0) | 40 (7.0) |  |
| **Best-corrected visual acuity into quartile (range)**** | | | |
| Q1 (-0.30, -0.08) | 273 (98.6) | 4 (1.4) | <0.001 |
| Q2 (0, 0.10) | 138 (92.0) | 12 (8.0) |  |
| Q3 (0.15, 0.82) | 133 (90.5) | 14 (9.5) |  |
| Q4 (0.83, 4.0) | 49 (77.8) | 14 (22.2) |  |
| Median (range) | 0.0 (-0.30, 3.0) | 0.3 (-0.18, 2.3) | <0.001 |
| **Lens status** | | | |
| Phakic | 576 (93.2) | 42 (6.8) | 0.528 |
| Aphakic | 0 (0) | 0 (0) |  |
| Pseudophakic | 17 (89.5) | 2 (9.5) |  |
| **Location of largest break** | | | |
| Superior | 366 (92.9) | 28 (7.1) | 0.801 |
| Inferior/posterior pole | 227 (93.4) | 16 (6.6) |  |
| **Size of largest break (degrees)** | | | |
| 0-30 | 565 (93.2) | 41 (6.8) | 0.533 |
| 30-60 | 22 (88.0) | 3 (12.0) |  |
| 60-90 | 3 (100) | 0 (0) |  |
| **Type of break** | | | |
| Hole | 326 (94.8) | 18 (5.2) | 0.071 |
| Tear | 267 (91.1) | 26 (8.9) |  |
| **PVR** | | | |
| PVR stage B | 20 (87.0) | 3 (13.0) | 0.237 |
| PVR stage N | 573 (93.3) | 41 (6.7) |  |
| **Surgical time into quartile (range)** | | | |
| Q1 (10, 51) | 129 (94.1) | 8 (5.9) | 0.735 |
| Q2 (52, 70) | 137 (94.5) | 8 (5.5) |  |
| Q3 (71, 97) | 144 (91.7) | 13 (8.3) |  |
| Q4 (98, 372) | 183 (92.5) | 15 (7.5) |  |
| Median(range) | 76 (20, 372) | 84 (35, 305) | 0.414 |
| **Drainage retinotomy** | | | |
| Performed | 3 (100) | 0 (0) | 0.637 |
| Not performed | 593 (93.1) | 44 (6.9) |  |
| **Intraoperative adjuvant use** | | | |
| Yes | 0 (0) | 0 (0) | - |
| No | 590 (93.1) | 44 (6.9) |  |
| **Intraoperative complications** | | | |
| Yes | 26 (92.9) | 2 (7.1) | 0.96 |
| No | 567 (93.1) | 42 (6.9) |  |

SB, scleral buckling; stage N, retinal detachment with stage A proliferative vitreoretinopathy (PVR) and no PVR

**P* values for categorical and continuous variables were obtained by chi-square test and Mann-Whitney U test, respectively.

**Decimal values were converted to the logarithm of the minimal angle of resolution (logMAR) units.
