# Supplementary material for: Effect of surgeon-related factors on outcome of retinal detachment surgery: analyses of data in Japan-retinal detachment registry
Source: Sci Rep. 2022 Mar 10;12:4213. doi: 10.1038/s41598-022-07838-5 (PMC8913601; doi:10.1038/s41598-022-07838-5)
Supplement: Supplementary file 3 — Supplementary Information 3. [file 41598_2022_7838_MOESM3_ESM.docx]

**Effect of Surgeon-Related Factors on Outcome of**

**Retinal Detachment Surgery: Analyses of Data**

**in Japan-Retinal Detachment Registry**

Keita Yamakiri^1,2^, Taiji Sakamoto^1,2^, Chihaya Koriyama^3^, Ryo Kawasaki ^2,4^, Takayuki Baba ^2,5^, Koichi Nishitsuka ^2,6^, Takashi Koto ^2,7^, Hiroto Terasaki ^1^ on behalf of Japan Retinal Detachment Registry

^1^Department of Ophthalmology, Kagoshima University Graduate School of Medical and Dental Sciences; ^2^The Japan-Retinal Detachment Registry Group; ^3^ Department of Epidemiology and Preventive Medicine, Kagoshima University Graduate School of Medical and Dental Sciences;^4^Department of Vision Informatics, Osaka University Graduate School of Medicine; ^5^Department of Ophthalmology, Chiba University; ^6^Department of Ophthalmology, Yamagata University; and ^7^Department of Ophthalmology, Kyorin Eye Center, Kyorin University School of Medicine.

| **Table　S3. Distributions of visual outcomes in PPV and SB cases.** (Online only) | | | | |
| --- | --- | --- | --- | --- |
|  | **No. of eyes (%)** | | |  |
|  | **Visual outcome in BCVA (logMAR)** | | | |
|  | **Improved** | **Unchanged** | **Worsened** | ***P* value** |
| All　(n=2,203) | 914 (41.5) | 986 (44.8) | 303 (13.8) |  |
| PPV (n=1,634) | 786 (48.1) | 663 (40.6) | 185 (11.3) | <0.001 |
| SB (n=569) | 128 (22.5) | 323 (56.8) | 118 (20.7) |  |
| Decimal values were converted to the logarithm of the minimal angle of resolution (logMAR) units. | | | | |
| Improvement in BCVA was defined as >0.2 difference in logMAR units in comparison between before and after the operation. Subjects whose visual acuity worsened after the operation were placed in "worsened" group. | | | | |
| The postoperative BCVA at 6 months was more frequently in PPV (48%) than in SB (22.4%) groups | | | | |

BCVA, best-corrected visual acuity; logMAR, logarithm of the minimal angle of resolution; PPV, pars plana vitrectomy; SB, scleral buckling.
