# Supplementary material for: Effect of surgeon-related factors on outcome of retinal detachment surgery: analyses of data in Japan-retinal detachment registry
Source: Sci Rep. 2022 Mar 10;12:4213. doi: 10.1038/s41598-022-07838-5 (PMC8913601; doi:10.1038/s41598-022-07838-5)
Supplement: Supplementary file 4 — Supplementary Information 4. [file 41598_2022_7838_MOESM4_ESM.docx]

**Effect of Surgeon-Related Factors on Outcome of**

**Retinal Detachment Surgery: Analyses of Data**

**in Japan-Retinal Detachment Registry**

Keita Yamakiri^1,2^, Taiji Sakamoto^1,2^, Chihaya Koriyama^3^, Ryo Kawasaki ^2,4^, Takayuki Baba ^2,5^, Koichi Nishitsuka ^2,6^, Takashi Koto ^2,7^, Hiroto Terasaki ^1^ on behalf of Japan Retinal Detachment Registry

^1^Department of Ophthalmology, Kagoshima University Graduate School of Medical and Dental Sciences; ^2^The Japan-Retinal Detachment Registry Group; ^3^ Department of Epidemiology and Preventive Medicine, Kagoshima University Graduate School of Medical and Dental Sciences;^4^Department of Vision Informatics, Osaka University Graduate School of Medicine; ^5^Department of Ophthalmology, Chiba University; ^6^Department of Ophthalmology, Yamagata University; and ^7^Department of Ophthalmology, Kyorin Eye Center, Kyorin University School of Medicine.

| **Table S4. Baseline characteristics in PPV cases of visual outcomes at 6 months.** (Online only) | | | | |
| --- | --- | --- | --- | --- |
| **Characteristics** | **No. of eyes (%)** | | | ***P* value** |
|  | Improved  (n=786) | Unchanged  (n=663) | Worsened  (n=185) |  |
| **Sex** | | | | |
| Female | 236 (43.6) | 240 (44.4) | 65 (12.0) | 0.037* |
| Male | 550 (50.3) | 423 (38.7) | 120 (11.0) |  |
| **Patient’s Age (years)** | | | | |
| <50 | 120 (47.2) | 106 (41.7) | 28 (11.0) | 0.082* |
| 50- | 245 (45.3) | 227 (42.0) | 69 (12.8) |  |
| 60- | 269 (47.4) | 241 (42.5) | 57 (10.1) |  |
| 70- | 152 (55.9) | 89 (32.7) | 31 (11.4) |  |
| Median (range) | 61 (17, 96) | 60 (20, 88) | 58 (33, 94) | 0.187*** |
| **Causes of retinal detachment** | | | | |
| Retinal tears related to traction | 693 (47.6) | 600 (41.2) | 163 (11.2) | 0.217* |
| Retinal holes, atrophic holes, or retinal atrophy with lattice degeneration | 64 (48.5) | 52 (39.4) | 16 (12.1) |  |
| Others | 29 (63.0) | 11 (23.9) | 6 (13.0) |  |
| **Status of macula** | | | | |
| Macula on | 600 (82.9) | 90 (12.4) | 34 (4.7) | <0.001** |
| Macula off | 178 (19.8) | 568 (63.3) | 151 (16.8) |  |
| Unknown | 8 (61.5) | 5 (38.5) | 0 (0.0) |  |
| **Previous ocular surgery** | | | | |
| Yes | 174 (46.9) | 143 (38.5) | 54 (14.6) | 0.079* |
| No | 612 (48.4) | 520 (41.2) | 131 (10.4) |  |
| **Best-corrected visual acuity into quartile (range)****** | | | | |
| Q1 (-0.30, -0.08) | 2 (0.5) | 298 (72.7) | 110 (26.8) | <0.001* |
| Q2 (0, 0.10) | 13 (3.9) | 279 (83.5) | 42 (12.6) |  |
| Q3 (0.15, 0.82) | 337 (75.6) | 80 (17.9) | 29 (6.5) |  |
| Q4 (0.83, 4.0) | 434 (97.8) | 6 (1.4) | 4 (0.9) |  |
| Median (range) | 1.0  (-0.08, 3.00) | 0.0  (-0.18, 1.52) | -0.1  (-0.30, 1.15) | <0.001*** |
| **Lens status** | | | | |
| Phakic | 644 (48.5) | 545 (41.0) | 140 (10.5) | 0.175** |
| Aphakic | 1 (25.0) | 2 (50.0) | 1 (25.0) |  |
| Pseudophakic | 141 (46.8) | 116 (38.5) | 44 (14.6) |  |
| **Location of largest break** | | | | |
| Superior | 610 (47.6) | 526 (41.0) | 146 (11.4) | 0.718* |
| Inferior/posterior pole | 176 (50.0) | 137 (38.9) | 39 (11.1) |  |
| **Size of largest break (degrees)** | | | | |
| 0-30 | 710 (47.8) | 615 (41.4) | 160 (10.8) | 0.024* |
| 30-60 | 68 (53.1) | 41 (32.0) | 19 (14.8) |  |
| 60-90 | 8 (38.1) | 7 (33.3) | 6 (28.6) |  |
| **Type of break** | | | | |
| Hole | 73 (50.0) | 56 (38.4) | 17 (11.6) | 0.848* |
| Tear | 713 (47.9) | 607 (40.8) | 168 (11.3) |  |
| **PVR** | | | | |
| PVR stage B | 47 (59.5) | 20 (25.3) | 12 (15.2) | 0.017* |
| PVR stage N | 739 (47.5) | 643 (41.4) | 173 (11.1) |  |
| **Surgical time into quartile(range)** | | | | |
| Q1 (10, 51) | 176 (42.7) | 198 (48.1) | 38 (9.2) | 0.007* |
| Q2 (52, 70) | 204 (47.6) | 177 (41.3) | 48 (11.2) |  |
| Q3 (71, 97) | 203 (49.0) | 158 (38.2) | 53 (12.8) |  |
| Q4 (98, 372) | 203 (53.6) | 130 (34.3) | 46 (12.1) |  |
| Median (range) | 72  (22, 223) | 65  (15, 186) | 76  (20, 300) | <0.001*** |
| **Drainage retinotomy** | | | | |
| Performed | 244 (52.0) | 163 (34.8) | 62 (13.2) | 0.010** |
| Not performed | 541 (46.5) | 500 (43.0) | 123 (10.6) |  |
| unknown | 1(100.0) | 0 (0.0) | 0 (0.0) |  |
| **Intraoperative adjuvant use** | | | | |
| Yes | 723 (49.1) | 586 (39.8) | 165 (11.2) | 0.063* |
| No | 63 (39.4) | 77 (48.1) | 20 (12.5) |  |
| **Intraoperative complications** | | | | |
| Yes | 128 (53.6) | 79 (33.1) | 32 (13.4) | 0.035* |
| No | 658 (47.2) | 584 (41.9) | 153 (11.0) |  |

PPV, pars plana vitrectomy; stage N, retinal detachment with stage A proliferative vitreoretinopathy (PVR) and no PVR

**P* values for categorized variables were obtained by chi-square test or **Fisher’s exact test.

*** Kruskal-Wallis test was used for the comparison of continuous variables.

****Decimal values were converted into the logarithm of the minimal angle of resolution (logMAR) units.
