# Supplementary material for: Effect of surgeon-related factors on outcome of retinal detachment surgery: analyses of data in Japan-retinal detachment registry
Source: Sci Rep. 2022 Mar 10;12:4213. doi: 10.1038/s41598-022-07838-5 (PMC8913601; doi:10.1038/s41598-022-07838-5)
Supplement: Supplementary file 5 — Supplementary Information 5. [file 41598_2022_7838_MOESM5_ESM.docx]

**Effect of Surgeon-Related Factors on Outcome of**

**Retinal Detachment Surgery: Analyses of Data**

**in Japan-Retinal Detachment Registry**

Keita Yamakiri^1,2^, Taiji Sakamoto^1,2^, Chihaya Koriyama^3^, Ryo Kawasaki ^2,4^, Takayuki Baba ^2,5^, Koichi Nishitsuka ^2,6^, Takashi Koto ^2,7^, Hiroto Terasaki ^1^ on behalf of Japan Retinal Detachment Registry

^1^Department of Ophthalmology, Kagoshima University Graduate School of Medical and Dental Sciences; ^2^The Japan-Retinal Detachment Registry Group; ^3^ Department of Epidemiology and Preventive Medicine, Kagoshima University Graduate School of Medical and Dental Sciences;^4^Department of Vision Informatics, Osaka University Graduate School of Medicine; ^5^Department of Ophthalmology, Chiba University; ^6^Department of Ophthalmology, Yamagata University; and ^7^Department of Ophthalmology, Kyorin Eye Center, Kyorin University School of Medicine.

| **Table S5. Baseline characteristics and visual outcomes at 6 months in cases that underwent SB surgery.** (Online only) | | | | |
| --- | --- | --- | --- | --- |
| **Characteristics** | **No. of eyes (%)** | | | ***P* value** |
|  | Improved  (n=128) | Unchanged  (n=323) | Worsened  (n=118) |  |
| **Sex** | | | | |
| Female | 54 (23.9) | 134 (59.3) | 38 (16.8) | 0.171* |
| Male | 74 (21.6) | 189 (55.1) | 80 (23.3) |  |
| **Patient’s Age (years)** | | | | |
| < 50 | 89 (25.1) | 208 (58.8) | 57 (16.1) | 0.007* |
| 50- | 20 (16.5) | 68 (56.2) | 33 (27.3) |  |
| 60- | 10 (14.9) | 36 (53.7) | 21 (31.3) |  |
| 70- | 9 (33.3) | 11 (40.7) | 7 (25.9) |  |
| Median (range) | 39 (14, 94) | 42 (14, 83) | 51 (11, 77) | 0.002*** |
| **Causes of retinal detachment** | | | | |
| Retinal tears related to traction | 60 (23.7) | 131 (51.8) | 62 (24.5) | 0.133* |
| Retinal holes, atrophic holes, or retinal atrophy with lattice degeneration | 65 (21.5) | 186 (61.4) | 52 (17.2) |  |
| Others | 3 (23.1) | 6 (46.2) | 4 (30.8) |  |
| **Status of macula** | | | | |
| Macula on | 109 (59.6) | 56 (30.6) | 18 (9.8) | <0.001** |
| Macula off | 18 (4.7) | 265 (69.2) | 100 (26.1) |  |
| Unknown | 1 (33.3) | 2 (66.7) | 0 (0) |  |
| **Previous ocular surgery** | | | | |
| Yes | 8 (13.8) | 30 (51.7) | 20 (34.5) | 0.015* |
| No | 120 (23.5) | 293 (57.3) | 98 (19.2) |  |
| **Best-corrected visual acuity into quartile (range)****** | | | | |
| Q1 (-0.30, -0.08) | 0 (0) | 171 (68.4) | 79 (31.6) | <0.001* |
| Q2 (0, 0.10) | 1 (0.7) | 108 (80.0) | 26 (19.3) |  |
| Q3 (0.15, 0.82) | 80 (62.0) | 38 (29.5) | 11 (8.5) |  |
| Q4 (0.83, 4.0) | 47 (85.5) | 6 (10.9) | 2 (3.6) |  |
| Median (range) | 0.70  (0.05, 3.0) | -0.08  (-0.18, 2.0) | -0.08  (-0.30, 1.10) | <0.001*** |
| **Lens status** | | | | |
| Phakic | 127 (23.1) | 316 (57.4) | 108 (19.6) | 0.002** |
| Pseudophakic | 1 (5.6) | 7 (38.9) | 10 (55.6) |  |
| **Location of largest break** | | | | |
| Superior | 77 (21.6) | 204 (57.3) | 75 (21.1) | 0.812* |
| Inferior/posterior pole | 51 (23.9) | 119 (55.9) | 43 (20.2) |  |
| **Size of largest break (degrees)** | | | | |
| 0-30 | 125 (23.0) | 309 (56.8) | 110 (20.2) | 0.511** |
| 30-60 | 3 (13.6) | 12 (54.6) | 7 (31.8) |  |
| 60-90 | 0 (0.0) | 2 (66.7) | 1 (33.3) |  |
| **Type of break** | | | | |
| Hole | 65 (21.7) | 183 (61.0) | 52 (17.3) | 0.057* |
| Tear | 63 (23.4) | 140 (52.0) | 66 (24.5) |  |
| **PVR** | | | | |
| PVR stage B | 6 (27.3) | 11 (50.0) | 5 (22.7) | 0.794* |
| PVR stage N | 122 (22.3) | 312 (57.0) | 113 (20.7) |  |
| **Surgical time into quartile (range)** | | | | |
| Q1 (10, 51) | 19 (15.3) | 80 (64.5) | 25 (20.2) | 0.075* |
| Q2 (52, 70) | 26 (18.8) | 80 (58.0) | 32 (23.2) |  |
| Q3 (71, 97) | 32 (24.6) | 67 (51.5) | 31 (23.9) |  |
| Q4 (98, 372) | 51 (28.8) | 96 (54.2) | 30 (17.0) |  |
| Median (range) | 85.5 (24, 305) | 72 (20, 212) | 74 (28, 229) | 0.007*** |
| **Drainage retinotomy** | | | | |
| Performed | 1 (50.0) | 1 (50.0) | 0 (0) | 0.678** |
| Not performed | 127 (22.4) | 322 (56.8) | 118 (20.8) |  |
| **Intraoperative adjuvant use** | | | | |
| Yes | 0 (0) | 0 (0) | 0 (0) |  |
| No | 128 (22.5) | 323 (56.8) | 118 (20.7) |  |
| **Intraoperative complications** | | | | |
| Yes | 7 (30.4) | 11 (47.8) | 5 (21.7) | 0.600* |
| No | 121 (22.2) | 312 (57.1) | 113 (20.7) |  |

SB, scleral buckling; stage N, retinal detachment with stage A proliferative vitreoretinopathy (PVR) and no PVR

**P* values for categorized variables were obtained by chi-square test or **Fisher’s exact test.

*** Kruskal-Wallis test was used for the comparison of continuous variables.

****Decimal values were converted into the logarithm of the minimal angle of resolution (logMAR) units.
