# Supplementary material for: Effect of surgeon-related factors on outcome of retinal detachment surgery: analyses of data in Japan-retinal detachment registry
Source: Sci Rep. 2022 Mar 10;12:4213. doi: 10.1038/s41598-022-07838-5 (PMC8913601; doi:10.1038/s41598-022-07838-5)
Supplement: Supplementary file 6 — Supplementary Information 6. [file 41598_2022_7838_MOESM6_ESM.docx]

**Effect of Surgeon-Related Factors on Outcome of**

**Retinal Detachment Surgery: Analyses of Data**

**in Japan-Retinal Detachment Registry**

Keita Yamakiri^1,2^, Taiji Sakamoto^1,2^, Chihaya Koriyama^3^, Ryo Kawasaki ^2,4^, Takayuki Baba ^2,5^, Koichi Nishitsuka ^2,6^, Takashi Koto ^2,7^, Hiroto Terasaki ^1^ on behalf of Japan Retinal Detachment Registry

^1^Department of Ophthalmology, Kagoshima University Graduate School of Medical and Dental Sciences; ^2^The Japan-Retinal Detachment Registry Group; ^3^ Department of Epidemiology and Preventive Medicine, Kagoshima University Graduate School of Medical and Dental Sciences;^4^Department of Vision Informatics, Osaka University Graduate School of Medicine; ^5^Department of Ophthalmology, Chiba University; ^6^Department of Ophthalmology, Yamagata University; and ^7^Department of Ophthalmology, Kyorin Eye Center, Kyorin University School of Medicine.

| **Table S6. Distributions and risk of visual outcomes by factors associated with surgeons’ experiences and activities in PPV cases.** (Online only) | | | | | | |
| --- | --- | --- | --- | --- | --- | --- |
|  | **Visual outcome: No. of eyes (%)** | | |  | **aOR (95%CI)**** | |
|  | **Improved** | **Unchanged** | **Worsened** | **P value*** | **Unchanged** | **Worsened** |
| **Number of total surgical experiences into quartile (range）** | | | | | |  |
| Q1 (4-90) | 193 (45.7) | 186 (44.1) | 43 (10.2) | 0.232 | reference | reference |
| Q2 (91-229) | 212 (53.1) | 140 (35.1) | 47 (11.8) |  | 0.98 (0.59, 1.63) | 1.75 (0.93, 3.31) |
| Q3 (230-460) | 203 (47.5) | 174 (40.8) | 50 (11.7) |  | 1.19 (0.72, 1.96) | 1.78 (0.95, 3.34) |
| Q4 (527-4574) | 178 (46.1) | 163 (42.2) | 45 (11.7) |  | 1.39 (0.80, 2.41) | 2.29 (1.15, 4.56) |
| Median (range) | 220.5 (4, 3,534) | 232 (4, 3,534) | 232 (26, 3,534) | 0.766 | P for trend = 0.189 | P for trend = 0.024 |
| **Duration of surgical experiences into quartile (range）** | | | | | |  |
| Q1 (1-3) | 185 (45.2) | 178 (43.5) | 46 (11.3) | 0.685 | reference | reference |
| Q2 (4-8) | 230 (48.6) | 194 (41.0) | 49 (10.4) |  | 0.89 (0.55, 1.45) | 0.94 (0.52, 1.71) |
| Q3 (9-14) | 178 (49.7) | 134 (37.4) | 46 (12.9) |  | 1.14 (0.65, 1.98) | 1.54 (0.80, 2.97) |
| Q4 (15-38) | 193 (49.0) | 157 (39.9) | 44 (11.2) |  | 1.10 (0.65, 1.87) | 1.46 (0.76, 2.81) |
| Median (range) | 8 (1, 38) | 8 (1, 38) | 8 (1, 38) | 0.346 | P for trend = 0.516 | P for trend = 0.108 |
| **Number of surgical experiences per year into quartile (range）** | | | | | |  |
| Q1 (2.1-20.1) | 221 (53.1) | 157 (37.7) | 38 (9.1) | 0.170 | reference | reference |
| Q2 (20.3-30) | 210 (48.8) | 167 (38.8) | 53 (12.3) |  | 1.25 (0.75, 2.07) | 1.77 (0.95, 3.31) |
| Q3 (30.2-45.1) | 183 (46.2) | 165 (41.7) | 48 (12.1) |  | 1.46 (0.87, 2.47) | 2.10 (1.10, 4.03) |
| Q4 (45.4-157.7) | 172 (43.9) | 174 (44.4) | 46 (11.7) |  | 1.22 (0.72, 2.04) | 1.57 (0.82, 3.00) |
| Median (range) | 29.5 (2.1, 136) | 30.3 (2.1, 136) | 30.2 (4.3, 136) | 0.014 | P for trend= 0.365 | P for trend= 0.159 |
| **Number of registered cases into quartile (range）** | | | | | |  |
| Q1 (1-28) | 191 (47.0) | 168 (41.4) | 47 (11.6) | 0.009 | reference | reference |
| Q2 (30-40) | 213 (51.1) | 144 (34.5) | 60 (14.4) |  | 1.10 (0.65, 1.85) | 1.78 (0.96, 3.31) |
| Q3 (41-63) | 204 (48.9) | 166 (39.8) | 47 (11.3) |  | 1.05 (0.63, 1.75) | 1.22 (0.65, 2.27) |
| Q4 (65-144) | 178 (45.2) | 185 (47.0) | 31 (7.9) |  | 0.98 (0.57, 1.67) | 0.71 (0.35, 1.40) |
| Median (range) | 40 (2, 122) | 43 (1, 122) | 39 (3, 122) | 0.040 | P for trend = 0.942 | P for trend = 0.249 |
| * Chi-square test or Kruskal-Wallis test. | | | |  |  |  |
| ** Adjusted odds ratio (aOR) for the risk of “unchanged” or “worsened” after the operation was estimated based on multinomial logistic regression analysis. This model was adjusted for sex, patient’s age, best corrected visual acuity, presence of macular detachment, and surgical time. | | | | | | |

PPV, pars plana vitrectomy; aOR, adjusted odds ratio; CI, confidence interval.
